# Interleukin-1 Receptor Antagonist Reduces Neonatal Lipopolysaccharide-Induced Long-Lasting Neurobehavioral Deficits and Dopaminergic Neuronal Injury in Adult Rats

**DOI:** 10.3390/ijms16048635

**Published:** 2015-04-17

**Authors:** Yi Pang, Lu-Tai Tien, Hobart Zhu, Juying Shen, Camilla F. Wright, Tembra K. Jones, Samir A. Mamoon, Abhay J. Bhatt, Zhengwei Cai, Lir-Wan Fan

**Affiliations:** 1 Department of Pediatrics, Division of Newborn Medicine, University of Mississippi Medical Center, Jackson, MS 39216, USA; E-Mails: ypang@umc.edu (Y.P.); zzhu@umc.edu (H.Z.); sunnylele@hotmail.com (J.S.); cfwright@umc.edu (C.F.W.); tkjones@umc.edu (T.K.J.); samamoon@umc.edu (S.A.M.); abhatt@umc.edu (A.J.B.); zcai@umc.edu (Z.C.); 2 School of Medicine, Fu Jen Catholic University, Xinzhuang Dist, New Taipei City 24205, Taiwan; E-Mail: 068154@mail.fju.edu.tw

**Keywords:** lipopolysaccharide, interleukin-1 receptor antagonist, dopaminergic neuronal injury, substantia nigra, microglia

## Abstract

Our previous study showed that a single lipopolysaccharide (LPS) treatment to neonatal rats could induce a long-lasting neuroinflammatory response and dopaminergic system injury late in life. This is evidenced by a sustained activation of microglia and elevated interleukin-1β (IL-1β) levels, as well as reduced tyrosine hydroxylase (TH) expression in the substantia nigra (SN) of P70 rat brain. The object of the current study was to test whether co-administration of IL-1 receptor antagonist (IL-1ra) protects against LPS-induced neurological dysfunction later in life. LPS (1 mg/kg) with or without IL-1ra (0.1 mg/kg), or sterile saline was injected intracerebrally into postnatal day 5 (P5) Sprague-Dawley male rat pups. Motor behavioral tests were carried out from P7 to P70 with subsequent examination of brain injury. Our results showed that neonatal administration of IL-1ra significantly attenuated LPS-induced motor behavioral deficits, loss of TH immunoreactive neurons, as well as microglia activation in the SN of P70 rats. These data suggest that IL-1β may play a pivotal role in mediating a chronic neuroinflammation status by a single LPS exposure in early postnatal life, and blockading IL-1β might be a novel approach to protect the dopaminergic system against perinatal infection/inflammation exposure.

## 1. Introduction

Increasing evidence suggests that perinatal infection is a risk factor for not only brain injury in newborns, but also certain neurological disorders developed later in life [1,2]. Elevated concentrations of proinflammatory cytokines, such as interleukin-1β (IL-1β), interleukin-6 (IL-6) and tumor necrosis factor-α (TNFα), are frequently detected in the brain [3,4], cord blood [5], and amniotic fluid [6] of very preterm infants with brain injury. Although these data strongly suggest a link between increased expression of proinflammatory cytokines and brain injury, a cause–effect relationship between cytokine induction and brain injury remains to be investigated.

Animal studies have shown that perinatal exposure to endotoxin lipopolysaccharide (LPS), a component of the cell wall of gram-negative bacteria [7], increases the risk for developing certain neurological disorders in animal models, such as Parkinson’s disease, schizophrenia, autism and cerebral palsy [8,9,10,11,12]. Our previous studies have shown that intracerebral injection of LPS to postnatal day 5 (P5) rats resulted in dopaminergic neuronal injury and neurobehavioral deficits at both neonatal and adult age [13,14,15]. The underlying mechanisms by which a single neonatal LPS exposure results in long-lasting adverse neurological effects are not clear; however, it appears that such exposure can trigger a chronic neuroinflammatory response, indicated by a persistent microglial activation and elevated interleukin-1β (IL-1β) expression in the substantia nigra (SN) of adult rats (P70) [15]. Based on these findings, we hypothesized that interleukin-1β (IL-1β) may play a central role in mediating a sustained neuroinflammation in our animal model.

IL-1 receptor antagonist (IL-1ra) is an endogenous cytokine which preferentially binds to the type-1 IL-1 receptor to block intracellular signaling, and injection of recombinant IL-1ra has the ability to inhibit actions of IL-1 [16]. Additionally, IL-1ra may also protect the brain by suppressing IL-1β production [17,18,19]. Therefore, blocking IL-1β activity by IL-1ra could be an effective approach to attenuate LPS-induced neurological disabilities. We previously reported that co-administration of LPS with IL-1ra via intracerebral (i.c.) injection provides protection against LPS-induced brain damage [20] and hyperalgesia [21] in neonatal rats. The aim of the current study is to determine whether IL-1ra provides long-lasting neuroprotection against neonatal LPS-induced chronic inflammation as well as dopaminergic neuronal injury in adult rats.

## 2. Results and Discussion

### 2.1. IL-1ra Attenuated Neurobehavioral Deficits Induced by Lipopolysaccharide (LPS) Exposure

**Figure 1:**
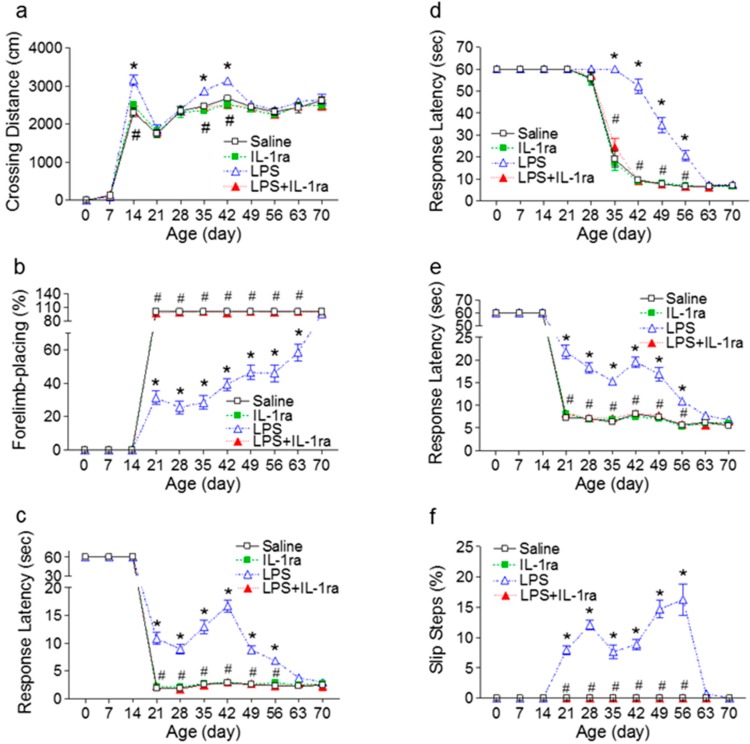
IL-1ra attenuated LPS-induced deficits in motor behaviors in rats from P7 to P70 rats. (**a**) Locomotor activity, as determined in the open field test; (**b**) The vibrissa-elicited forelimb-placing test; (**c**) The movement initiation test; (**d**) The pole test; and (**e**,**f**) The tapered/ledged beam walking test as determined by slip step ratio (**e**) and performance latency (**f**). The results are expressed as the mean ± SEM of 12 animals in each group, and analyzed by two-way repeated measures ANOVA for data from tests conducted continuously at different postnatal days. * *p* < 0.05 represents significant difference for the LPS group as compared with the saline group on the same postnatal day. # *p* < 0.05 represents significant difference for the LPS+IL-1ra group as compared with the LPS group on the same postnatal day.

First we tested whether IL-1ra could ameliorate LPS-induced neurobehavioral deficits in rats. Several motor behavioral tests, including locomotion, vibrissa-elicited forelimb-placing, movement initiation, pole test, and tapered/ledged beam walking test, are sensitive tools to assess dopaminergic system maturation during postnatal development in rodents. Neonatal LPS injection resulted in several motor behavioral deficits in LPS-exposed rats at both juvenile and adolescent stages. Interestingly, those behavioral deficits were spontaneously reversible, and by P70 there were no significant differences of motor behavior between LPS-exposed and the control rats (Figure 1). IL-1ra treatment significantly attenuated LPS-induced motor behavioral impairment (Figures 1). This suggests that IL-1β plays a pivotal role in LPS-induced adverse effects on neurodevelopment.

#### 2.1.1. Locomotion

The total crossing distance, which measures horizontal locomotor activity, was significantly longer in LPS-treated rats as compared to the controls (F(3, 527) = 12.994, *p* < 0.001) on P14, P35 and P42 (*p* < 0.05) (Figure 1a). IL-1ra treatment prevented LPS-induced hyperactivity in the LPS+IL-1ra group (*p* < 0.05) (Figure 1a).

#### 2.1.2. Vibrissa-Elicited Forelimb-Placing Test

All rats from the control group succeeded in vibrissa-elicited forelimb-placing test (100%) from P21 (Figure 1b). The success rate of the vibrissa-elicited forelimb-placing test in the LPS-injected group was significantly lower than that of the control group (F(3, 527) = 787.903, *p <* 0.001) from P21 to P63 (*p <* 0.05) (Figure 1b). IL-1ra treatment significantly improved LPS-induced lower success rate of the vibrissa-elicited forelimb-placing test (*p <* 0.05) (Figure 1b).

#### 2.1.3. Movement Initiation Test

Rats from the control group initiated stepping movements in 1~4 s period for one forelimb in a balanced order (Figure 1c) from P21. The neonatal LPS-exposed rats took longer time to initiate a movement (F(3, 527) = 267.267, *p <* 0.001) between P21 to P56 (*p <* 0.05) (Figure 1c), indicating the forelimb akinesia (prolongation of the reaction time). IL-1ra treatment prevented the LPS-induced prolongation of initiated stepping movements in the LPS+IL-1ra group (*p <* 0.05) (Figure 1c).

#### 2.1.4. Pole Test

The performance latency of pole test in the neonatal LPS-injected group was longer than that of the control group (F(3, 527) = 203.913, *p <* 0.001) from P28 to P56 (*p <* 0.05) (Figure 1d). The LPS-induced bradykinesia (prolongation of the movement time) was prevented by treatment with IL-1ra (*p <* 0.05) (Figure 1d).

#### 2.1.5. Tapered/Ledged Beam Walking Test

All P21 and older rats from the control group succeeded in the tapered/ledged beam walking test in less than 10 s with no foot-faults (slips) made with the hindlimbs, which were measured as an index of hindlimb function (Figure 1e,f). The neonatal LPS-injected group needed a longer time to complete the task (beam walking latency) than that of the control (F(3, 527) = 206.970, *p <* 0.001) from P21 to P56 (*p <* 0.05) (Figure 1e). The neonatal LPS-exposed rats also made more foot-faults (slips) with the hindlimbs (F(3, 527) = 408.750, *p <* 0.001) from P21 to P56 (*p <* 0.05) (Figure 1f). IL-1ra treatment prevented the LPS-induced behavioral deficits on the tapered/ledged beam walking test (*p <* 0.05) (Figure 1e,f).

### 2.2. IL-1ra Attenuated Neonatal LPS Exposure-Induced Losses of TH-Immunoreactive Neurons

**Figure 2:**
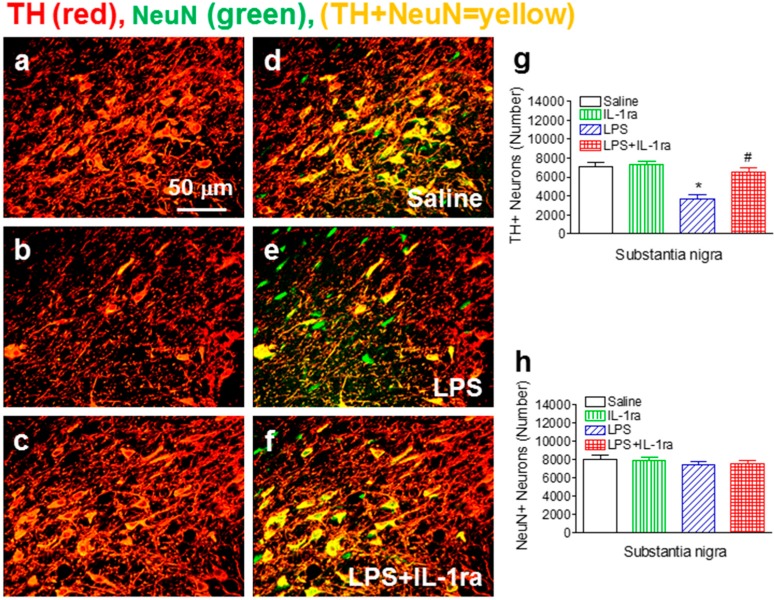
IL-1ra attenuated neonatal LPS-induced reduction on the number of TH+ cells in the SN of P70 rat brain. Representative photomicrographs of TH+ (red color) (**a**,**b**,**c**); NeuN+ (green color) cells, or double-labeled cells (yellow) (**d**,**e**,**f**) in the SN areas of the midbrain sections; (**a**,**d**) saline-treated group; (**b**,**e**) LPS-treated group; (**c**,**f**) LPS+IL-1ra group; Scale bar: 40 μm. Stereological quantification of TH+ cells (**g**); and NeuN+ cells (**h**) in the SN area were shown in **g** and **h**, respectively. The results are expressed as the mean ± SEM of six animals in each group, and analyzed by two-way ANOVA. * *p* < 0.05 represents a significant difference for the LPS group as compared with the saline group. # *p* < 0.05 represents significant difference for the LPS+IL-1ra group as compared with the LPS group.

Next, we examined the integrity of dopaminergic neurons in the rat SN by TH immunostaining. The total number of TH+ neurons was assessed by an unbiased stereological approach. Consistent with our previous report [15], the total number of TH+ dopaminergic neurons in the SN of P70 rat brain was significantly reduced by neonatal LPS exposure (F(1, 23) = 28.632, *p <* 0.001) (*p <* 0.05) (Figure 2b,e,g), while the total number of NeuN+ neurons in the SN was not affected (F(1, 23) = 1.742, *p* = 0.202, ns) (Figure 2e,h), as compared to the control group (Figure 2a,d,g,h). IL-1ra treatment significantly attenuated the reduction of TH+ cell number (F(1, 23) = 11.198, *p* = 0.003) (*p <* 0.05) (Figure 2c,f,g). These data suggest that there was no actual loss of dopaminergic neurons in the SN of LPS-exposed rats; rather, such exposure resulted in a long-lasting effect on the phenotype of dopaminergic neurons. In Parkinson’s patients, initial signs of motor impairment appear when 50%–70% of nigrostriatal dopaminergic neurons have been lost [22,23]. In the current study, neonatal LPS exposure caused a 47% decrease of TH+ dopaminergic neurons but only an 8% decrease of NeuN+ neurons in the SN at P70 (Figure 2), suggesting that there was no real loss of dopaminergic neurons but rather a reduced TH expression. This is consistent with the behavioral data which showed there were no significant differences of motor behavior between LPS-exposed rats and the control rats at P70 (Figure 1). The reduced TH immunoreactivity might reflect a silent neurotoxicity, presumably caused by a sustained neuroinflammation with critical involvement of IL-1β signaling. We have previously shown that LPS-induced TNFα protein was detected as early 2 h; however, it was non-detectable after 48 h. In contrast, the induction of IL-1β expression was relatively delayed but remained elevated weeks after LPS injection [24]. It is known that IL-1β can strongly activate microglia to release more IL-1β, which in turn activates more microglia. This may be a potential mechanism underlying chronic neuroinflammation. Since SN contains the highest density of microglia in the brain while dopaminergic neurons are uniquely susceptible to injury, a single neonatal LPS challenge may initiate a chronic neuroinflammation that are mostly prominent in the SN region, leading to a silent neurotoxicity to dopaminergic neurons during development. In our animal model, rats treated with IL-1ra showed significant improvement in motor behavior performance and protection of TH+ cells in the SN, suggesting that IL-1β is critically involved in chronic neuroinflammation-induced neuronal injury of the dopaminergic system.

### 2.3. IL-1ra Attenuated Neonatal LPS Exposure-Induced Loss of Dopaminergic Dendrites and Reduction in Mitochondrial Complex I Activity

**Figure 3:**
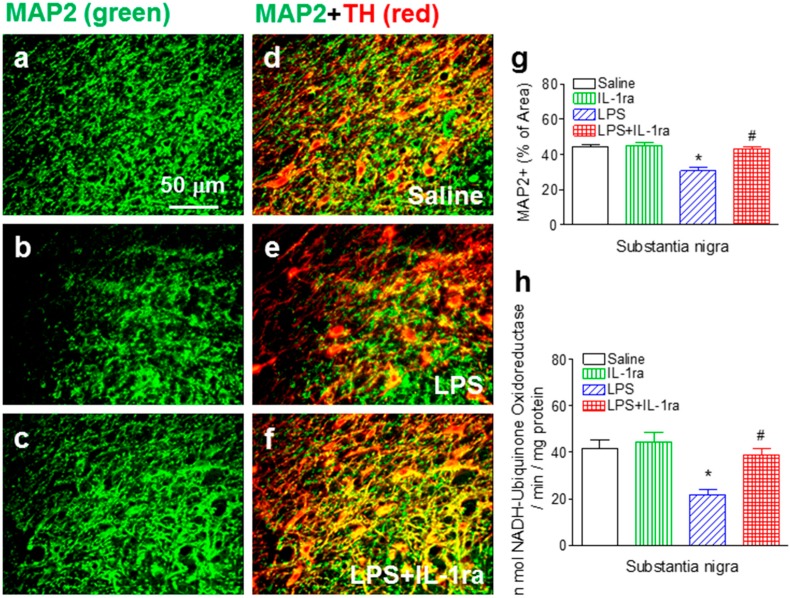
IL-1ra attenuated neonatal LPS-induced dendritic damage and reduction in mitochondrial complex I activity in the SN area of the P70 rat brain. Representative photomicrographs of MAP2+ (green color) (**a**–**c**); TH+ (red color) cells; or double-labeled cells (yellow) (**d**–**f**); (**a**,**d**) saline-treated group; (**b**,**e**) LPS-treated group; (**c**,**f**) LPS+IL-1ra group. Scale bar: 40 μm; (**g**) Quantitation of the percentage area of image that contained MAP2+ staining in the SN of P70 rat; (**h**) Quantitation of the enzymatic activity of mitochondrial complex I in the SN of P70 rat. The results are expressed as the mean ± SEM of six animals in each group, and analyzed by two-way ANOVA. * *p* < 0.05 represents significant difference for the LPS group as compared with the saline group. # *p* < 0.05 represents significant difference for the LPS+IL-1ra group as compared with the LPS group.

The reduction of TH expression without cell loss suggest possible functional impairment of dopaminergic neurons. To test this notion, we examined the dendrites and mitochondrial complex I activity in the SN area of P70 rats. Our quantitative MAP2+ immunostaining data showed that neonatal LPS exposure reduced the dendritic density (F(1, 23) = 21.625, *p* < 0.001) (*p* < 0.05) (Figure 3b,e,g) as compared to that in the control group (Figure 3a,d,g), as well as mitochondrial complex I activity in the SN area of the P70 rat brain (F(1, 23) = 13.866, *p* = 0.001) (*p* < 0.05) (Figure 3h). Treatment with IL-1ra significantly attenuated reduction in the dendritic density (F(1, 23) = 12.277, *p* = 0.002) (*p* < 0.05) (Figure 3c,f,g) and mitochondrial complex I activity in the SN area of the P70 rat brain (F(1, 23) = 4.601, *p* = 0.044) (*p* < 0.05) (Figure 3h).

### 2.4. IL-1ra Suppressed Chronic Microglia Activation Following LPS Exposure

**Figure 4:**
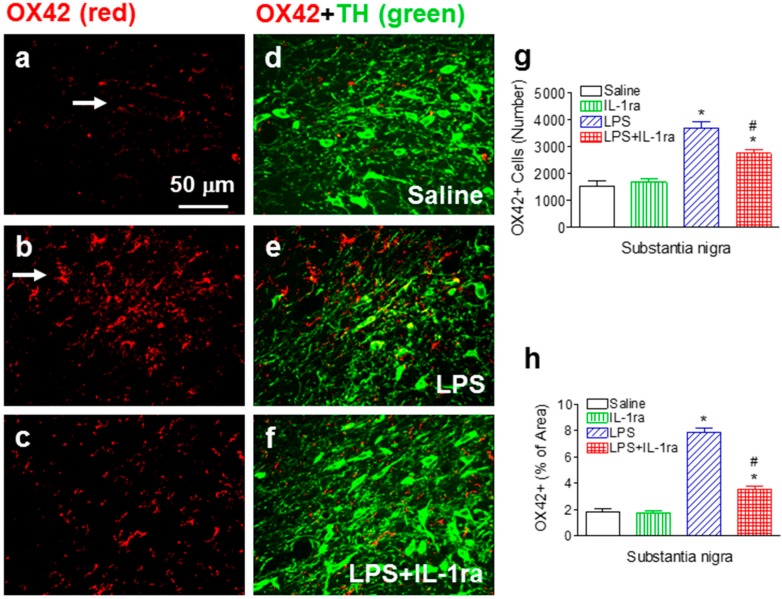
IL-1ra attenuated neonatal LPS-induced microglia activation in the SN area of the P70 rat brain. Representative photomicrographs of OX42+ (red color) (**a**–**c**); TH+ (green color) cells, or the merged double staining images (**d**–**f**) in the rat brain; (**a**,**d**) saline-treated group; (**b**,**e**) LPS-treated group; (**c**,**f**) LPS+IL-1ra group. Scale bar: 40 μm; Microglia activation was quantified by counting the number of OX42+ cells in the SN (**g**) or calculating the percentage area of OX42+ staining in the digitally acquired images from the SN (**h**). The results are expressed as the mean ± SEM of six animals in each group, and analyzed by two-way ANOVA. * *p* < 0.05 represents significant difference for the LPS group as compared with the saline group. # *p* < 0.05 represents significant difference for the LPS+IL-1ra group as compared with the LPS group.

Neonatal LPS exposure resulted in a sustained microglial activation indicated by an increase in numbers as well as features of activated morphology of OX42+ cells. In the control rat brain, the few OX42+ cells that were detected exhibited resting status characterized by smaller rod-shaped soma with fine and ramified processes (Figure 4a,d, red). In contrast, significantly increased numbers of OX42+ cells were found in the SN area of the LPS-exposed rat brain (F(1, 23) = 81.252, *p <* 0.001) (*p <* 0.05) (Figure 4b,e,g). Moreover, the majority of these OX42+ cells showed typical features of activated microglia, namely bright staining of an elongated or round-shaped cell body with blunt or no processes [25] (indicated by arrows in Figure 4b). Higher percentage of OX42+ immunostaining area was observed in SN of the LPS-exposed rat brain (F(1, 23) = 226.422, *p <* 0.001) (*p <* 0.05) (Figure 4h). IL-1ra significantly suppressed LPS-induced microglia activation, as shown by reduced number of OX42+ cells (F(1, 23) = 9.543, *p* = 0.0061) (*p <* 0.05) (Figure 4c,f,g) as well as the percentage area of OX42+ immunostaining in the SN area of P70 rat (F(1, 23) = 64.638, *p <* 0.001) (*p <* 0.05) (Figures 4h).

### 2.5. IL-1ra Significantly Reduced Proinflammatory Cytokine Production Following LPS Exposure

**Figure 5:**
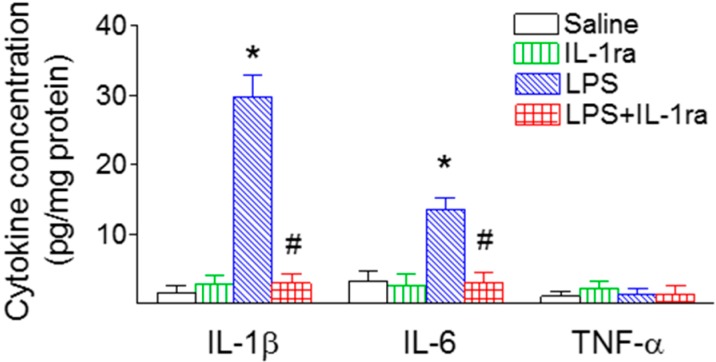
IL-1ra significantly attenuated induction of proinflammatory cytokines in the SN of P70 rat brain following LPS exposure. IL-1β, IL-6 and TNFα concentrations were determined by ELISA, as described in the Methods section. The results are expressed as the mean ± SEM of six animals in the unit of pg/mg protein in each group, and analyzed by two-way ANOVA. * *p <* 0.05 represents significant difference for the LPS group as compared with the saline group. # *p <* 0.05 represents significant difference for the LPS+IL-1ra group as compared with the LPS group.

LPS induced a robust inflammatory response in the rat SN, as shown by a strong induction of proinflammatory cytokines (Figure 5). Two major proinflammatory cytokines, IL-1β and IL-6 were undetectable in the saline-injected rat brain, but were significantly increased in the SN area of the P70 rat brain (IL-1β, F(1, 23) = 52.246, *p* < 0.001; IL-6, F(1, 23) = 12.311, *p* = 0.002) (*p* < 0.05) (Figure 5). In contrast, TNFα was undetectable in the SN region of the P70 control or LPS-exposed rat brain (F(1, 23) = 0.0530, *p* = 0.820) (Figure 5). Co-administration of LPS with IL-1ra reduced LPS exposure-induced elevation of IL-1β (F(1, 23) = 50.299, *p* < 0.001) (*p* < 0.05) and IL-6 levels in the SN region at P70 (F(1, 23) = 10.655, *p* = 0.004) (*p* < 0.05) (Figure 5).

### 2.6. IL-1ra Attenuated Enhanced Locomotor Activity in LPS-Exposed Rats Following Methamphetamine Stimulation

**Figure 6:**
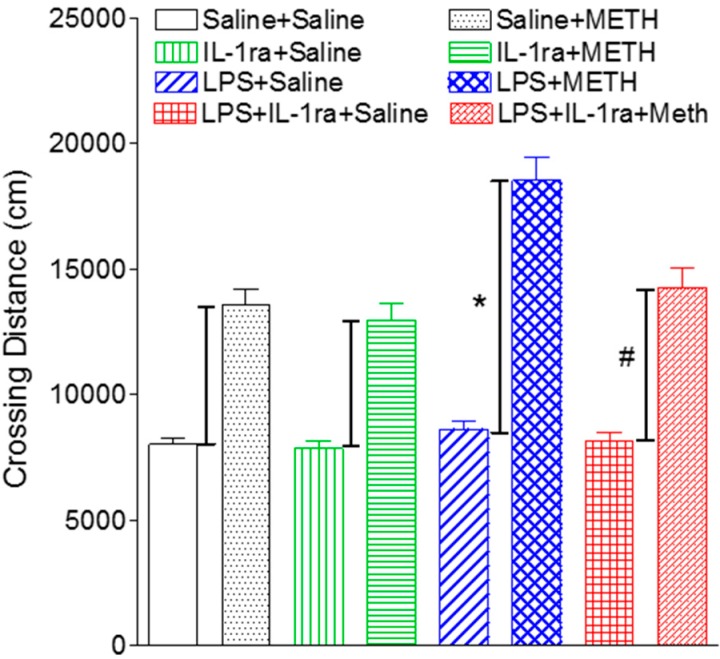
IL-1ra significantly attenuated enhanced locomotor activity (total distance traveled) upon METH challenge in P70 rats following neonatal LPS exposure. The results are expressed as the mean ± SEM of six animals in each group and analyzed by two-way ANOVA. * *p <* 0.05 represents significant difference between the increment in METH-increased locomotor activity within the LPS group (∆_(LPS+METH – LPS+Saline)_) as compared to that within the saline group (∆_(Saline+METH − Saline+Saline)_). # *p <* 0.05 represents significant difference between the increment in METH-increased locomotor activity within the LPS+IL-1ra group (∆_(LPS-IL-1ra+METH − LPS-IL-1ra+Saline)_) as compared to that within the LPS group (∆_(LPS+METH − LPS+Saline)_).

Consistent with our previous data [15], the above results indicate that while neonatal LPS exposure-induced motor neurobehavioral deficits were spontaneously recovered when rats reached adulthood (Figure 1), brain injury to the dopaminergic system as well as chronic inflammation persisted (Figure 2, Figure 3, Figure 4 and Figure 5). To further quantify functional integrity of the dopaminergic system, methamphetamine (METH, 0.5 mg/kg), which promotes the release of dopamine resulting in increases in motor activity [26], was administered subcutaneously to P70 rats. LPS-exposed rats showed enhanced locomotor activity upon METH challenge (F(1, 23) = 30.122, *p <* 0.001) (*p <* 0.05) (Figure 6), suggesting that neonatal LPS exposure has a long-lasting impact on the functional integrity of dopaminergic system. This is most likely due to a chronic neuroinflammation which might be associated with a silent neurotoxicity [15,27,28]. Our data showed that IL-1ra treatment significantly attenuated the enhanced locomotor activity in LPS-exposed rats upon METH in P70 rats (F(1, 23) = 12.517, *p* = 0.002) (*p <* 0.05) (Figure 6), further supporting the notion that IL-1β plays a critical role in mediating a chronic neuroinflammatory status, which could lead to compromise to the dopaminergic system.

The mechanisms by which neuroinflammation leads to the dopaminergic system injury is not fully understood; however, several components of the dopaminergic system, including striatal dopaminergic terminals, central dopamine turnover, as well as dopaminergic receptor sensitization, could be affected since alterations of any of these components have been shown to underlie enhanced behavioral reaction upon METH administration in the animals [26,28]. The enhanced locomotion in LPS-exposed rats upon METH challenge represents functional alterations in the dopaminergic system, although motor functions are normal for the time-being. Thus, a chronic neuroinflammation is associated with a silent neurotoxicity, which may be exacerbated upon a second hit. This concept has been introduced recently as a “double-hit” theory to explain why certain neurodegenerative disorders have a possible fetal origin [27]. The theory is supported from animal studies which demonstrate that animals predisposed to perinatal infection/inflammation were especially vulnerable to a second environmental insult at adult age [10,27,28]. One of the possible mechanisms underlying the “silent neurotoxicity” may relate to dendritic mitochondrial function. It has been reported that normal neurobehavioral performance depends directly on the degree of dendritic arborization and synaptic connectivity [29]. In addition to the reduction in the number of TH+ cells in the SN and the ventral tegmental area (VTA) of P6, P21 and P70 rats [13,14,15,30], neonatal LPS exposure also produced the damage on the dendrites and axons in both SN and VTA regions of P6, P21, and P70 [15,30,31]. Without METH challenge, LPS-treated animals with partially impaired SN dendritic mitochondrial function may not exhibit motor behavioral deficits. When challenged with METH, however, the already injured dendritic mitochondria become incapable to deal with the additional stress so that abnormal motor responses become apparent. The present data showed that IL-1ra treatment not only prevented the LPS-induced dendritic damage and reduction in mitochondrial complex I activity in the SN area (Figure 3), but also attenuated enhanced behavioral reaction upon METH in LPS-treated rats at P70 (Figure 3), suggesting IL-1β is involved in a silent neurotoxicity.

IL-1β is mainly produced by microglia upon LPS treatment [32]. The largest population of newborn microglia emerges in late gestation and early postnatal period in both humans and rats [33,34,35,36]. Our previous studies have shown that neonatal LPS exposure induced brain microglia activation and IL-1β expression, which persisted from P6, P14, to at least P21 [13,20,24,37]. Therefore, LPS exposure at neonatal stage can dramatically induce brain inflammatory responses and possibly primes microglia. Since IL-1β itself can activate microglia, it is possible that there is a feedback loop between IL-1β and microglia activation. This may explain why blocking IL-1β significantly suppressed the chronic neuroinflammation in this animal model.

**Figure 7:**
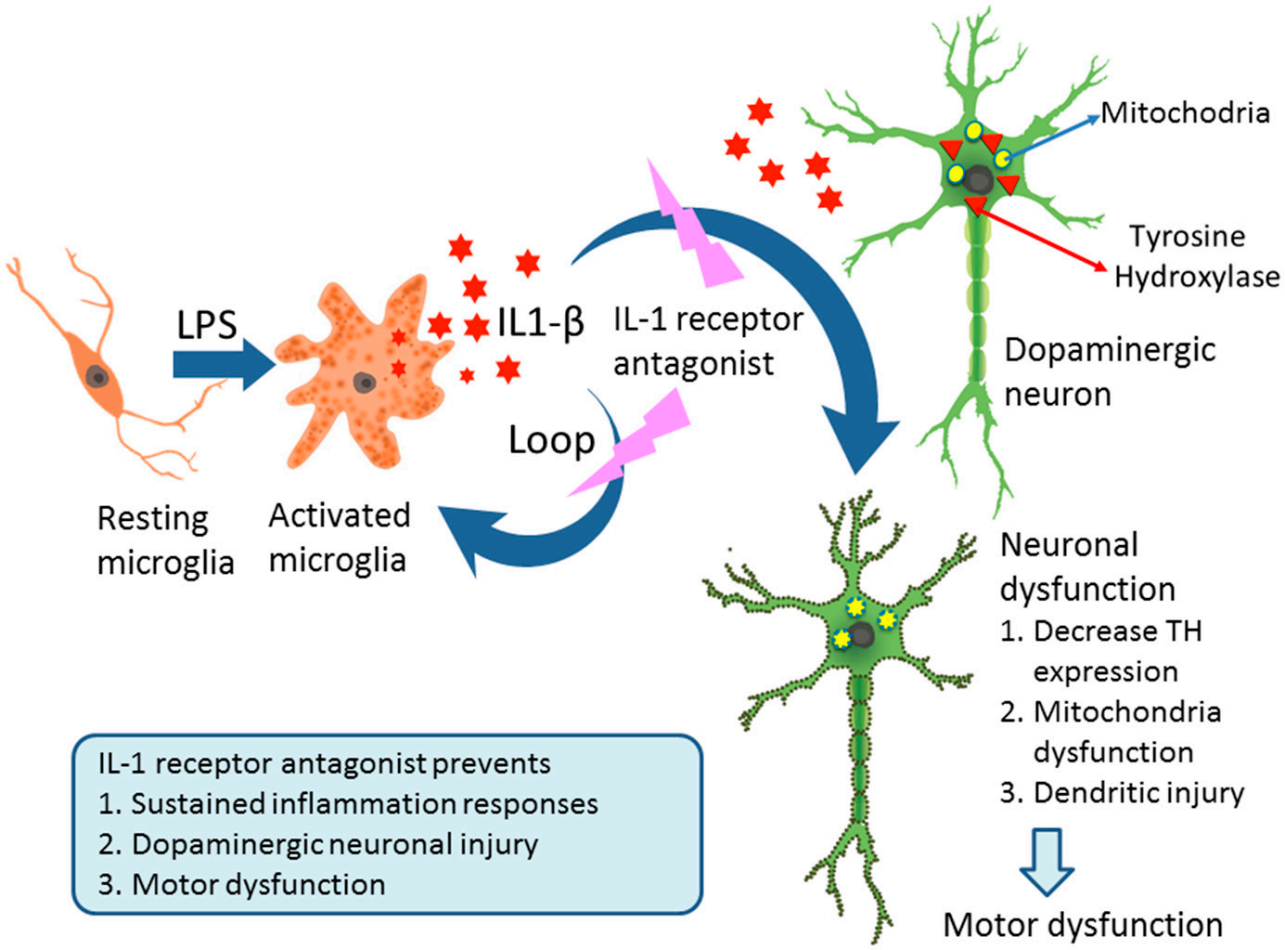
A schematic illustration of the possible mechanisms that underlie chronic neuroinflammation, dopaminergic neuronal injury, and motor behavioral disturbance in LPS-exposed rats at neonatal age. These compromises in both neuronal structures and neurobehaviors were attenuated by IL-1ra, suggesting that IL-1β plays a critical role in mediating these pathological changes.

In summary (Figure 7), neonatal LPS exposure induced a chronic neuroinflammation and a silent neurotoxicity, including microglia activation, elevated brain IL-1β contents, loss of TH+ immunoreactivity, dendritic damage and deduction in mitochondrial complex I activity in the dopaminergic neurons in the SN, and disturbances of motor behaviors of P70 rats. Treatment with IL-1ra not only reduced neuroinflammation, but also provided protection against LPS-induced dopaminergic neuronal injury, suggesting IL-1β plays a central role in inflammatory neuronal injury in this animal model. This information implies that approaches to block IL-1β may be a potential new treatment for dopaminergic neuronal injury induced by infection/inflammation.

## 3. Experimental Section

### 3.1. Chemicals

Unless otherwise stated, all chemicals used in this study were purchased from Sigma (St. Louis, MO, USA). Recombinant rat IL-1ra was purchased from R&D Systems (Minneapolis, MN, USA). Monoclonal mouse antibodies against neuron-specific nuclear protein (NeuN), or microtubule-associated protein 2 (MAP2); and OX42 (CD11b) were purchased from Millipore (Billerica, MA, USA) and Serotec (Raleigh, NC, USA), respectively. Polyclonal rabbit antibody against tyrosine hydroxylase (TH) was purchased from Millipore. ELISA kits for immunoassay of rat IL-1β (RLB00), interleukin-6 (IL-6) (R6000B) and tumor necrosis factor-α (TNFα) (RTA00) were purchased from R&D Systems.

### 3.2. Animals

Pregnant Sprague-Dawley rats arrived in the laboratory at day 19 of gestation. Animals were maintained in a room on a 12 h light/dark cycle at constant temperature (22 ± 2 °C). The day of birth was defined as postnatal day 0 (P0). The litter size was adjusted to 12 pups per litter with pups being weaned on P21. All procedures for animal care were conducted in accordance with the Guide for the Care and Use of Laboratory Animals of the National Institutes of Health, and in addition, to the Animal Care and Use Committees at the University of Mississippi Medical Center and Fu Jen Catholic University of Taiwan.

### 3.3. Surgical Procedures and Animal Treatment

In order to eliminate a possible gender difference, only male rats were used in the present study. Intracerebral injection of LPS, or LPS in combination with IL-1ra in 5-day-old male Sprague-Dawley rat pups was performed as previously described [15,20,21]. Under light anesthesia with isoflurane (1.5%~5%), LPS (1 mg/kg from *Escherichia coli*, serotype 055:B5), IL-1ra (0.1 mg/kg), or LPS (1 mg/kg) plus IL-1ra (0.1 mg/kg) in sterile saline containing 0.1% bovine serum albumin (BSA, total volume 2 μL) was administered into the rat brain (1.0 mm posterior to and 1.0 mm left of bregma, and 2.0 mm deep from the skull surface) by using a stereotaxic apparatus with a neonatal rat adapter. The injection site was located in the area just above the left cingulum. The control rats were injected with the same volume of sterile saline containing 0.1% BSA. The dose of LPS used here was based on our previous procedures [15,20,21]. Our previous studies have shown that neonatal LPS (1 mg/kg) exposure induces long-lasting neurobehavioral impairment and dopaminergic neuronal injury in adult rats [15,21]. The dose of IL-1ra was chosen based on the data on peak concentrations of IL-1β achieved in the rat pup brain following LPS administration, as reported previously [24,38]. All animals survived the intracerebral injection procedure.

Each dam had the same litter size (12 pups including 6 males and 6 females) and equal numbers of saline, saline plus IL-1ra, LPS, and LPS plus IL-1ra-treated rat pups were included in a litter. The pups were weaned at P21 and male rats were housed four per cage after weaning (on cage each for saline, saline plus IL-1ra, LPS, and LPS plus IL-1ra-treated rats). The female rats were used for another study. There were 120 rats (30 rats from each group) from 20 litters that were used in the present study. In each group, 12 were used for behavioral tests from P7 to P70. Six rats from each group were sacrificed by transcardiac perfusion with normal saline followed by 4% paraformaldehyde for brain section preparation. These rats were used for preparation of free-floating coronal brain sections at 40 μm of thickness in a sliding microtome (SM 2000R; Leica, Wetzlar, Germany). The sections then processed with immunohistochemistry staining and stereological estimates of the total number of neurons in the SN. For determination of the content of pro-inflammatory cytokines or mitochondrial complex I activity in the SN, 12 P70 rats from each group were sacrificed by decapitation to collect fresh brain tissue. The rest of the 12 rats from the saline, saline plus IL-1ra, LPS, and LPS plus IL-1ra-treated group were further divided into two groups: one received a subcutaneous (s.c.) injection of methamphetamine (METH) (0.5 mg/kg) (6 rats for each group) and the other of saline (6 rats for each group) on P70.

### 3.4. Behavioral Testing

The behavioral tests were performed by an investigator blind to the treatment as described previously [10,15] with modifications. The developmental test battery used was based on the tests for neurobehavioral toxicity [39,40,41]. Behavioral tests including the locomotion (distance traveled), vibrissa-elicited forelimb-placing test, movement initiation test, pole test, and tapered/ledged beam walking test were used to determine motor functions. All animals were tested in the same order once a week from P7 to P70.

#### 3.4.1. Locomotor Activity

The open field test measures the activity and habituation responses of animals upon placement in a novel environment [40]. Locomotor activity was measured using the Any-Maze™ Video Tracking System (Stoelting Co., Wood Dale, IL, USA). Animals were placed in the activity chamber (42 cm × 25 cm × 40 cm) in a quiet room with dimmed light. The total distance traveled by the animal was recorded during a 10 min testing period [10,15].

#### 3.4.2. Vibrissa-Elicited Forelimb-Placing Test

The test measures forelimb placing upon stimulation of the rat’s vibrissae, which is an effective tool to assess the integrity of nigrostriatal system [42,43,44]. Briefly, the testing rat was gently held by its torso and turned sideways, allowing the vibrissae to be perpendicular to the surface of the table. The vibrissae were brushed against the edge of a table, and the percentage of successful replacement of the contralateral forelimb onto the tabletop was recorded for each side. Each experiment consisted of 10 trials. Typically, intact animals place their forelimbs quickly onto the counter top with a 100% of success rate.

#### 3.4.3. Movement Initiation Test

Movement initiation for each forelimb was assessed to test the forelimb akinesia [43,45,46]. The animal was held by its torso with its hindlimbs and one forelimb lifted above the surface of the table so that the weight of the animal’s body was supported by one forelimb alone. The animal was allowed to initiate stepping movements in a 60 s period for one forelimb and then the other in a balanced order. The time to initiate one step was recorded for each forelimb, and initiation times for both forelimbs were averaged to create one score.

#### 3.4.4. Pole Test

This test assesses the maturation of ascending and descending skills of developing rat pups [39]. The rat pup was placed on a pole (diameter: 3 cm, length: 50 cm) facing upward. A corkball was installed at the top of the pole, so that the testing rat pup faced a choice of either going upwards or downwards. The test consisted of three trials per week. The performance latency of experimental animals is the total time spent to turn around and to reach the platform at the bottom of the pole (cutoff time: 60 s).

#### 3.4.5. Tapered/Ledged Beam Walking Test

Sensorimotor functions of forelimbs and hindlimbs were tested using a tapered/ledged beam [43,47]. Foot faults (slips) made with the hindlimbs can be measured as an index of hindlimb function. The tapered/ledged beam consists of the staging area (6 cm × 15 cm), trapezoid walking beam (6 and 1.5 cm wide and 135 cm long) and an off-loading area (1.5 cm × 15 cm). The entire apparatus was elevated to a height of 50 cm above the floor. Rats were allowed to transverse a tapered beam with underhanging ledges on each side to permit foot faults without falling. The rats’ performance was visually recorded and later analyzed by calculating the slip ratio of the hindlimb (number of slips/number of total steps). The time spent on the beam for each animal that traversed the beam was recorded. The slip ratios for both hindlimbs were averaged to create one score. The mean of three trails was used for statistical analyses.

#### 3.4.6. Methamphetamine-Induced Locomotion

Locomotor activity was measured in the activity chamber as described above. On P70 experimental day, rats were placed in the chambers 1 h before the administration of saline or METH to acclimatize them to their surroundings. Baseline levels of locomotion were determined during the 10 min prior to METH or saline administration (−10 to 0 min). After rats received saline or METH (0.5 mg/kg, s.c.), their activities were recorded for 1 h.

### 3.5. Immunohistochemistry Studies

Primary antibodies were used in the following dilutions: NeuN (1:200), TH (1:1000), MAP2 (1:200) and OX42 (1:200). NeuN detects the neuron-specific nuclear protein which primarily localizes in the nucleus of the neurons with slight staining in the cytoplasm. TH was used to detect dopaminergic neurons in the SN. MAP2 provides selective staining of neuronal dendritic processes. Microglia were detected using OX42 immunostaining, which recognizes both the resting and the activated microglia. Sections were incubated with primary antibodies at 4 °C overnight and further incubated with secondary antibodies conjugated with fluorescent dyes (Cy2, 1:100 or Cy3, 1:300; Jackson Immunoresearch, West Grove, PA, USA) for 1 h in the dark at room temperature. 4',6-Diamidine-2-phenylindole (DAPI, 100 ng/mL) was used simultaneously to stain nuclei in order to aid their identification during the final visualization. Sections incubated in the absence of a primary antibody were used as negative controls. The resulting sections were examined under a fluorescent microscope (BX60; Olympus, Center Valley, PA, USA) at appropriate wavelengths.

### 3.6. Stereological Estimates of the Total Number of Neurons in the Substantia Nigra (SN)

The stereological estimates of the total number of TH+ and NeuN+ neurons (*est N*) were performed in the SN of P70 rat brain, following the methods described by Ling *et al.* [12] and Lokkegaard *et al.* [48]. Nine equally spaced thick sections (40 μm) in the midbrain level that were to be used in the analysis came from a one-in-six series. The total number of TH+ or NeuN+ cells (*est N*) were counted in each of the nine sections, which cover the entire SN region. We found in our previous study that FITC-labeled LPS administered through a unilateral i.c. injection distributed quickly to both the hemispheres within 0.5 h (data not shown) and that unilaterally injected LPS caused similar enlargement of both lateral ventricles [20]. Furthermore, following a unilateral i.c. injection of LPS or saline in the P5 rat brain, no significant differences in density of TH+ and NeuN+ cells were observed between the ipsilateral (left) and the contralateral side (right) in the P21 rat brain [14]. Therefore, stereological cell counting in the present study was performed in the ipsilateral side of the rat brain. Stereological cell counting in the present study was focused in the SN region (nucleus A9 cells or cells in both the substatia nigra pars compacta and the substantia nigra pars reticulata). The Cavalieri principle [49] was used to estimate the reference volumes, *est* *V*(*ref*), and the volume density, *est N_V_*. The product of the two is an estimate of total number of cells in this region: *est V(ref)* X *est N_V_ = est N* [12,48,50].

### 3.7. Determination of Mitochondrial Complex I Activity

Complex I activity was determined by a spectrophotometric assay based on the quantification of the rate of oxidation of the complex I substrate NADH to ubiquinone as described by Champy *et al.* [51] and Hoglinger *et al.* [52] with minor modifications. SN tissues from each rat were collected 65 days after LPS injection (P70). Tissues were isolated, frozen in liquid nitrogen, and stored at −80 °C. The frozen brain tissue was homogenized mechanically, sonicated on ice in 10 mM Tris-HCl buffer (pH 7.2) containing 225 mM mannitol, 75 mM saccharose and 0.1 mM EDTA, and then centrifuged (600× *g*) for 20 min at 4 °C, to obtain post-nuclear supernatants. The optical density of the supernatants (40 μg sample protein) in 1 mL of an assay mixture was spectrophotometrically recorded at a wavelength of 340 nm for 200 s at 37 °C. The assay mixture was a potassium phosphate buffer (25 mM, pH 7.5) containing 2 mM potassium cyanide, 5 mM magnesium chloride, 2.5 mg/mL bovine serum albumin, 2 μM antimycin A, 100 μM decylubiquinone and 300 μM NADH. The proportion of NADH oxidation sensitive to an excess of rotenone (10 μM) was attributed to the complex I. This procedure minimizes the dissociation of rotenone from the complex I because of the use of small buffer volumes, maintenance at low temperatures and rapid analysis. The specific activity (nmol NADH oxidation/min/mg protein) of Complex I (NADH-ubiquinone oxidoreductase) was calculated using a molar extinction coefficient ε_340nm_ = 6.22 mM^−1^·cm^−1^ [53]. Enzyme activities were expressed as nmol/min/mg of brain tissue. (Complex I activity = (Rate (min^−1^)/ε_340nm_ (6.22 mM^−1^·cm^−1^))/0.040 mg).

### 3.8. Determination of IL-1β, IL6 and TNFα Protein by ELISA

Three major proinflammatory cytokines, IL-1β, IL-6 and TNFα, were determined by ELISA as previously described [15,20,38]. Briefly, rats were sacrificed by a decapitation and the fresh SN tissues from each rat were collected 65 days after LPS injection (P70). Tissues were homogenized by sonication in 1 mL ice-cold PBS (pH 7.2) and centrifuged at 12,000× *g* for 20 min at 4 °C. The supernatant was collected and the protein concentration was determined by the Bradford method [54]. ELISA was performed following manufacturer’s instructions and data were acquired using a 96-well plate reader (BioTek Instruments, Inc., Winooski, VT, USA). The cytokine contents were expressed as pg cytokines/mg protein.

### 3.9. Quantification of Immunostaining Data

The total number of OX42 positive cells (*est N*) were estimated in the SN using the stereological approach as described previously. As an alternative approach to assess microglia activation, the percentage area that contained OX42 positive staining to the entire captured image was calculated by software, as described previously [15]. In addition to cell counting, the density of OX42 or MAP2 immunoreactivity was also quantified by this method in the current study, according to published methods [10,14,15].

### 3.10. Statistics

The behavioral data were presented as the mean ± SEM and analyzed by the two-way repeated measures analysis of variance (ANOVA), which is suitable for data from tests conducted continuously at different postnatal days or by two-way ANOVA for METH-induced locomotion, followed by the Student-Newman-Keuls test. Results with a *p <* 0.05 were considered statistically significant. Data from stereological cell counting, immunostaining, mitochondrial complex I activity, and ELISA were presented as the mean ± SEM and analyzed by two-way ANOVA followed by Student-Newman-Keuls test. Results with a *p <* 0.05 were considered statistically significant.

## 4. Conclusions

Neonatal LPS exposure resulted in neurobehavioral deficits and dopaminergic neuronal injury associated with persistent neuroinflammation. IL-1ra treatment significantly attenuated LPS-induced chronic neuroinflammation, dopaminergic neuronal injury, and disturbances of motor behaviors in adult rats (Figure 7). Our findings suggest that the blockage of IL-1β might be an effective treatment for dopaminergic neuronal injury induced by early infection/inflammation.
